# Complete pathological response to neoadjuvant chemoradiotherapy in Crohn’s disease–associated fistula adenocarcinoma: a case report

**DOI:** 10.3389/fmed.2026.1746760

**Published:** 2026-04-01

**Authors:** Wafa Dahmeni, Emna Souilem, Houssem Hassen, Yossr Ghdiri, Zayen Ahlem, Ahmed Dahmoul, Aida Ben Slama, Ahlem Brahem, Hanen Jaziri, Sarra Mestiri, Nour Elleuch, Sihem Hmissa, Mehdi Ksiaa

**Affiliations:** 1 Department of Gastroenterology, Sahloul University Hospital, Sousse, Tunisia; 2 Department of Radiology, Sahloul University Hospital, Sousse, Tunisia; 3 Department of Pathology, Sahloul University Hospital, Sousse, Tunisia

**Keywords:** case report, complete pathological response, Crohn’s disease, fistula-associated adenocarcinoma, neoadjuvant chemoradiotherapy

## Abstract

Fistula-associated adenocarcinoma (FAA) in Crohn’s disease is an exceptionally rare and aggressive complication, often diagnosed late and historically managed with immediate radical surgery. Critically, the role of neoadjuvant chemoradiotherapy has remained undefined, with no reported cases of complete pathological response to date. We report the case of a 52-year-old man with longstanding Crohn’s disease who developed a mucinous adenocarcinoma arising from a chronic perianal fistula, initially revealed by a perineal abscess. Histopathological analysis confirmed the diagnosis. Staging work-up demonstrated no evidence of distant metastasis. The patient underwent neoadjuvant chemoradiotherapy according to the PRODIGE-23 protocol, followed by abdominoperineal resection. Pathological assessment of the resection specimen demonstrated a complete pathological response (ypT0N0). This case highlights the importance of maintaining high clinical suspicion for malignant transformation in patients with chronic perianal fistulas in Crohn’s disease and provides the first evidence that complete pathological response is achievable in fistula-associated adenocarcinoma, suggesting that neoadjuvant multimodal therapy may allow exploration of organ-preserving approaches in this rare condition.

## Introduction

Chronic perianal fistulas affect up to 90% of Crohn’s disease (CD) patients with colonic involvement and represent a particularly challenging aspect of disease management (1). While perianal fistulas are common, malignant transformation is extremely rare but clinically significant event (2). Diagnosis is frequently delayed due to nonspecific symptoms that mimic benign fistulas (3, 4). Abdominoperineal resection (APR) remains the cornerstone of curative treatment (4, 5). However, the role and efficacy of neoadjuvant chemoradiotherapy (CRT) in fistula-associated adenocarcinoma have not been well established, and no cases of complete pathological response have been previously reported.

This report describes the first documented case of complete pathological response to neoadjuvant chemoradiotherapy for CD-associated fistula adenocarcinoma, suggesting a potential paradigm shift in managing this devastating complication.

## Case report

A 52-year-old non-smoking man with a 30-year history of stenosing ileocolic CD presented with new-onset perineal pain and a draining perianal swelling.

He had been diagnosed with CD at the age of 22 after a moderate inflammatory flare, treated with corticosteroids and maintained on 5-aminosalicylic acid (5-ASA). In 2007, he experienced acute intestinal obstruction requiring ileocecal resection. Surveillance colonoscopy seven months later revealed endoscopic recurrence, prompting initiation of azathioprine (2 mg/kg/day). He subsequently achieved sustained clinical and endoscopic remission, allowing discontinuation of azathioprine in 2013 after five years of treatment.

Perianal disease first appeared in 2014 as a low intersphincteric simple fistula, initially managed by antibiotics with initial clinical improvement. Intermittent minor symptoms persisted over the following years. In early 2024, he presented again with progressive perineal pain and a tender, draining swelling in the left perianal region. He denied changes in bowel habits, bleeding, or weight loss. Examination in the genu-pectoral position revealed a 4-cm erythematous, warm, fluctuant swelling at the 7 o’clock position, with a productive external fistulous opening. Digital rectal examination detected no palpable anal canal or distal rectal masses.

The initial diagnosis was a perianal abscess complicating a chronic fistula. The patient received antibiotics followed by abscess drainage and fistulectomy. Postoperative recovery was uneventful. However, histopathological analysis of the excised specimen unexpectedly revealed mucinous adenocarcinoma arising within the fistulous tract.

**FIGURE 1:**
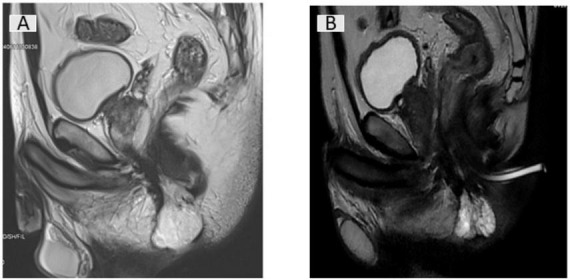
Sagittal T2-weighted pelvic MRI before **(A)** and after **(B)** chemoradiotherapy.

Subsequent colonoscopy showed no endoluminal lesions and confirmed the absence of active luminal Crohn’s disease. Pelvic magnetic resonance imaging (MRI) demonstrated a 42 mm × 37 mm × 21 mm oblong lesion in the left sublevator perianal region, communicating with the intergluteal cleft and associated with a complex fistulous tract showing irregular peripheral enhancement (Figure 1A). Thoracoabdominal computed tomography excluded distant metastases.

Given the fistula origin and absence of active Crohn’s disease, the tumor was managed as low rectal cancer. Neoadjuvant treatment consisted of six cycles of FOLFOX chemotherapy followed by pelvic radiotherapy (50.4 Gy in 25 fractions) with concurrent capecitabine, according to the PRODIGE-23 protocol.

**FIGURE 2:**
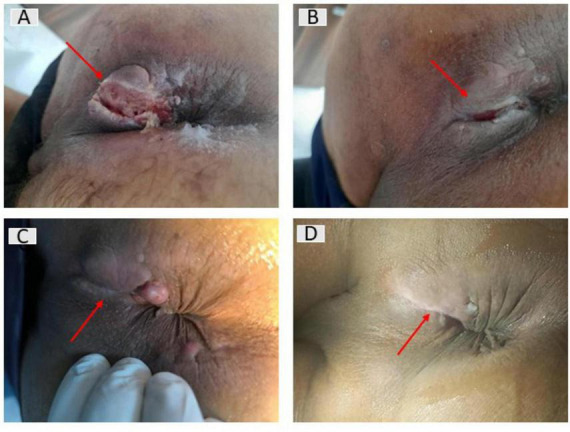
Evolution of perianal mucinous adenocarcinoma across chemoradiotherapy. **(A)** Initial presentation. **(B)** Early post-chemoradiotherapy appearance, demonstrating marked reduction of inflammatory signs, decreased discharge, and partial epithelialization of the external opening appearance at completion of therapy. **(C)** Progressive regression, characterized by collapse of the fistulous tract and residual fibrotic induration without active suppuration. **(D)** Final clinical aspect before surgery, showing complete closure of the external opening, replaced by a fibrotic scar with mild skin thickening, consistent with clinical response.

Clinical reassessment after chemoradiotherapy showed resolution of the productive fistulous opening, replaced by a fibrotic scar with mild skin thickening (Figure 2). Follow-up pelvic MRI two months after radiotherapy demonstrated marked regression of the sublevator mass, which appeared as a small cystic lesion (30 mm × 22 mm × 10 mm) with necrotic changes and minimal enhancement. The complex fistulous network had simplified to a single residual tract (Figure 1B).

Colonoscopy and CT imaging remained negative for residual or metastatic disease. The patient subsequently underwent abdominoperineal resection with permanent colostomy.

**FIGURE 3:**
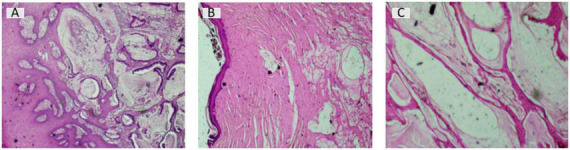
Histopathological response after chemoradiotherapy for mucinous adenocarcinoma arising from a chronic anal fistula. **(A)** Pretreatment biopsy showing mucinous adenocarcinoma composed of malignant glandular structures and abundant extracellular mucin (Hematoxylin and Eosin stain, ×40). **(B)** Surgical specimen after chemoradiotherapy showing extensive extracellular acellular mucin within fibrotic tissue (H&E, ×100). **(C)** High-power field of the resected specimen confirming acellular mucin lakes without viable tumor cells (H&E, ×200).

Histopathological examination of the surgical specimen demonstrated a complete pathological response: the tumor bed was entirely replaced by acellular mucin pools and dense fibrosis, with no viable carcinoma identified. Surgical margins were clear, and all five lymph nodes were negative for metastasis. The final stage was ypT0N0Mx, with a Mandard tumor regression grade (TRG) of 1 (Figure 3).

**FIGURE 4:**
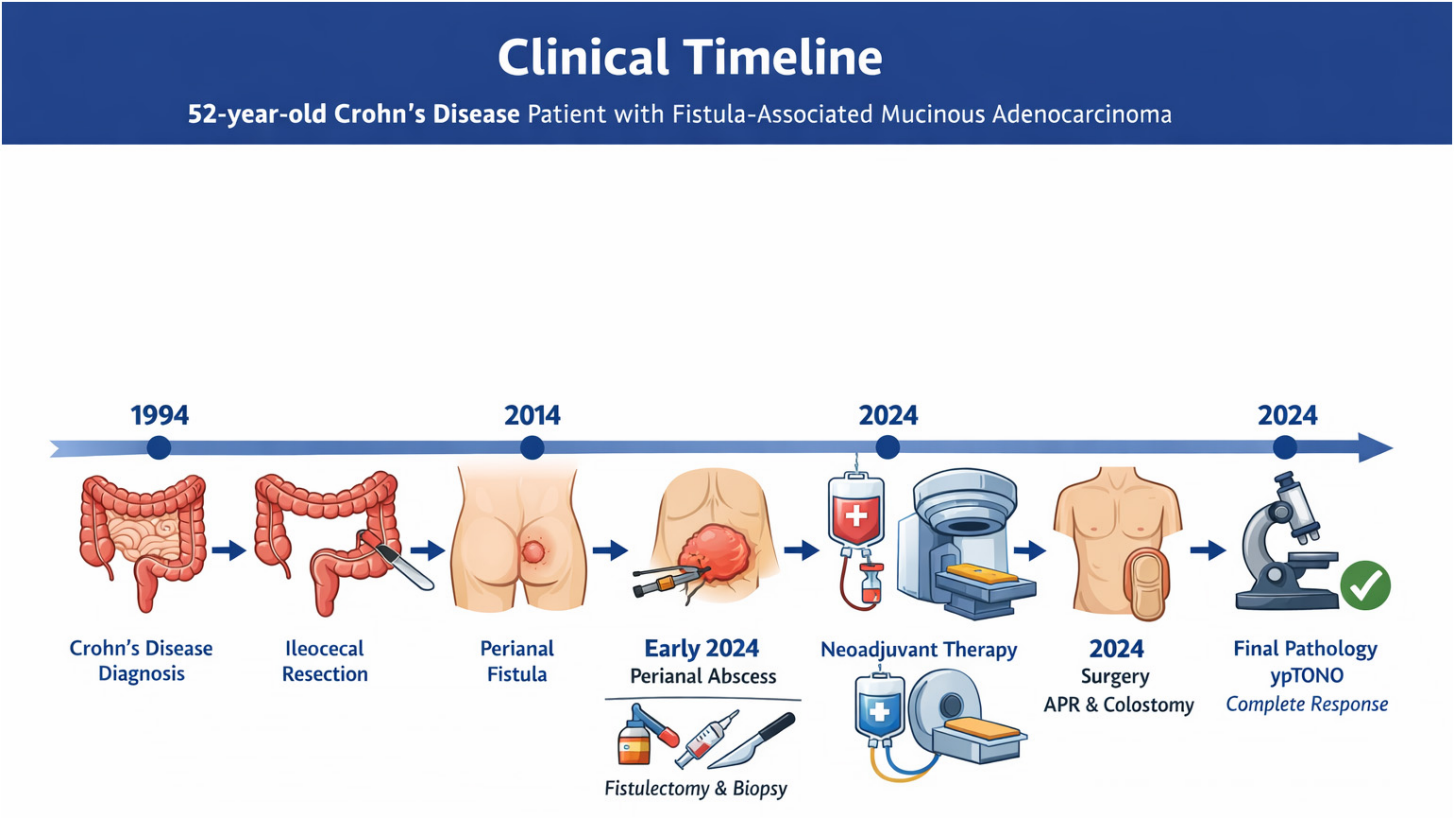
Chronological clinical timeline of the patient’s disease course.

A schematic timeline summarizing the patient’s clinical course, interventions, and outcomes is presented in Figure 4.

## Patient perspective

The patient reported significant symptom relief after neoadjuvant chemoradiotherapy, satisfaction with the multidisciplinary care, and improved quality of life. She highlighted the importance of clear communication with the medical team and expressed hope that sharing her case may help others.

## Discussion

Since its first description by 6, malignancy arising from Crohn’s disease–associated perianal fistulas has been recognized as a potential complication of the disease (7–9). Despite decades of clinical observation, the true incidence of fistula-associated adenocarcinoma remains poorly defined due to the rarity of this complication, with current estimates suggesting an occurrence rate of less than 0.7% among CD patients with perianal disease (2, 10–13). The pathogenesis of malignant transformation in chronic fistulas is multifactorial, involving persistent inflammation, impaired wound healing, and repeated cycles of mucosal destruction and regeneration, all contributing to a microenvironment conducive to neoplastic progression (1, 7, 14–16). Several risk factors have been consistently identified, including early disease onset, prolonged disease duration, and the presence of long-standing perianal fistulas (13, 15, 17). On average, 20–25 years elapse from CD diagnosis to cancer development (2, 13), and 6–15 years from fistula onset to malignancy (1, 5, 17). These timelines align with our case, in which the fistula appeared 17 years after CD diagnosis and malignant transformation occurred 11 years later.

Adenocarcinoma, most frequently of the mucinous subtype, represents the predominant histological pattern of fistula-associated malignancy, whereas squamous cell carcinoma is less commonly observed (2, 18, 19). Clinical presentation is often nonspecific and largely overlaps with benign fistulizing Crohn’s disease, contributing to diagnostic delay (3, 4). Commonly reported symptoms include purulent discharge, recurrent abscesses, rectal bleeding, and perineal pain (5, 7, 9, 15). For this reason, clinicians must maintain a high index of suspicion in patients with long-standing or complex fistulas, particularly when symptoms become refractory to conventional therapy, show progressive worsening, or newly arise after periods of clinical stability (4, 5, 20).

Diagnosis remains challenging and relies on a multimodal approach. Histopathological analysis is the gold standard; however, sampling limitations contribute to high initial false-negative rates, reported between 26% and 40%. These limitations often necessitate repeat biopsies and, in many cases, require sampling from multiple sites along the fistula tract (4, 21). Given these diagnostic challenges, imaging plays a crucial complementary role. Pelvic MRI serves as the primary imaging modality for evaluating suspected malignant transformation. While MRI can identify concerning features such as mass formation, irregular tract thickening, and heterogeneous contrast enhancement patterns, its diagnostic sensitivity remains limited due to the inherent difficulty in distinguishing between benign inflammatory changes and early malignant transformation (2, 11, 22).

Management strategies are not standardized due to the rarity of this cancer. Surgical resection, typically abdominoperineal resection (APR), remains the cornerstone of curative treatment (4, 5, 15, 23). However, achieving complete resection can be challenging, as highlighted by the high rates of positive margins reported in the literature (23). The role of neoadjuvant chemoradiotherapy (CRT) has evolved, although mucinous adenocarcinomas are traditionally considered less responsive to CRT than conventional adenocarcinomas. Nevertheless, emerging evidence suggests potential benefit from multimodal treatment. Recent case series have demonstrated improved local control and survival with aggressive multimodal treatment strategies in fistula-associated anal adenocarcinoma. Gaertner et al. (8) reported favorable long-term outcomes following radical surgery with perioperative chemoradiotherapy in a cohort of 14 patients. Similarly, Hongo et al. (24) observed a significantly higher rate of negative resection margins in patients treated with neoadjuvant radiotherapy or chemoradiotherapy compared with surgery alone, without evidence of pathological complete response. A review by de Souza et al. (21) demonstrated improved survival outcomes with combined neoadjuvant CRT and surgery compared to surgery alone.

In this case, the use of neoadjuvant CRT protocols established for locally advanced rectal cancer resulted in a complete pathological response, with clear margins and excellent local control. To our knowledge, this is the first documented report of complete pathological response using this specific regimen for mucinous adenocarcinoma arising from a Crohn’s-related fistula, highlighting the potential value of multimodal therapy in selected patients.

Given the rarity of fistula-associated adenocarcinoma, standardized management guidelines are lacking, and further research is urgently needed. The promising response observed in our case supports the evaluation of neoadjuvant CRT in larger, multicenter cohorts to better define optimal treatment parameters and identify predictors of response. Advances in imaging and molecular diagnostics may also help facilitate earlier detection of malignant transformation in patients with long-standing or complex fistulas.

## Conclusion

This case illustrates the potential of neoadjuvant chemoradiotherapy to achieve a complete pathological response in mucinous adenocarcinoma arising from a perianal fistula in Crohn’s disease. While radical surgical resection remains the cornerstone of treatment, our findings suggest that multimodal therapy may represent a valuable strategy in carefully selected patients with this rare malignancy. Larger prospective studies are needed to validate these observations and to establish evidence-based treatment algorithms for fistula-associated adenocarcinoma in Crohn’s disease.

## Strengths of the case report

1. Rarity and originality: The case reports a rare fistula-associated adenocarcinoma in Crohn’s disease achieving complete pathological response (pCR) after neoadjuvant chemoradiotherapy, which has not been previously documented.
2. Detailed clinical description: Provides a comprehensive timeline of presentation, diagnosis, therapeutic interventions, and outcomes, in line with CARE guidelines.

## Limitations of the case report

1. Single-case nature: Findings cannot be generalized and do not provide definitive guidance for treatment strategies such as organ preservation.
2. Limited follow-up: The follow-up period may be insufficient to assess long-term outcomes or recurrence.

## Data Availability

The original contributions presented in this study are included in the article/supplementary material, further inquiries can be directed to the corresponding author.
